# A historical, evidence-based, and narrative review on commonly used dietary supplements in lipid-lowering

**DOI:** 10.1016/j.jlr.2023.100493

**Published:** 2023-12-23

**Authors:** Jelani K. Grant, Michael Dangl, Chiadi E. Ndumele, Erin D. Michos, Seth S. Martin

**Affiliations:** 1 Ciccarone Center for the Prevention of Cardiovascular Disease, Division of Cardiology, Johns Hopkins University School of Medicine, Baltimore, MD, USA; 2 Internal Medicine Department, University of Miami Miller School of Medicine/ Jackson Memorial Hospital, Miami, FL, USA

**Keywords:** dietary supplements, fish oil, supplements, lipids, inflammation, lipid lowering

## Abstract

Dietary supplements augment the nutritional value of everyday food intake and originate from the historical practices of ancient Egyptian (Ebers papyrus), Chinese (Pen Ts’ao by Shen Nung), Indian (Ayurveda), Greek (Hippocrates), and Arabic herbalists. In modern-day medicine, the use of dietary supplements continues to increase in popularity with greater than 50% of the US population reporting taking supplements. To further compound this trend, many patients believe that dietary supplements are equally or more effective than evidence-based therapies for lipoprotein and lipid-lowering. Supplements such as red yeast rice, omega-3 fatty acids, garlic, cinnamon, plant sterols, and turmeric are marketed to and believed by consumers to promote “cholesterol health.” However, these supplements are not subjected to the same manufacturing scrutiny by the Food and Drug Administration as pharmaceutical drugs and as such, the exact contents and level of ingredients in each of these may vary. Furthermore, supplements do not have to demonstrate efficacy or safety before being marketed. The holistic approach to lowering atherosclerotic cardiovascular disease risk makes dietary supplements an attractive option to many patients; however, their use should not come at the expense of established therapies with proven benefits. In this narrative review, we provide a historical and evidence-based approach to the use of some dietary supplements in lipoprotein and lipid-lowering and provide a framework for managing patient expectations.

A dietary supplement is defined as any product that is intended to augment the nutritional value of everyday food intake, including vitamins, minerals, herbs, botanicals, and amino acids (1). Regulated similar to food, dietary supplements are not intended to treat, diagnose, cure or alleviate disease. Using data from the National Health and Nutrition Examination Survey, the sale of dietary supplements accounts for an estimated 50 billion dollars in the United States in 2020 with more than 50% of adults reporting any supplement use (2). Furthermore, between 2007–2008 and 2017–2018 dietary supplement use increased from 48.6% to 56.1% (3). Possible explanations for this increasing popularity include that supplement use is thought to be more “natural” and allays concerns regarding adverse reactions of pharmaceutical drugs, is perceived to align more closely with the ideologies of patients, satisfies a desire for more personalized and self-administered healthcare and is sold without the need for a drug prescription.

In tandem with the observed increase in supplement use, the prevalence of atherosclerotic cardiovascular disease (ASCVD) continues to rise from 2010–2020 (4), and multisociety guidelines emphasize the importance of evidence-based preventive therapies such as statin and nonstatin therapy targeting LDL-C lowering (5, 6). The increasing prevalence of ASCVD is thought to be due to both a growing and aging population combined with inadequate cardiovascular risk factor control (7). In the absence of high-quality data, the increasing use of unproven dietary supplements for LDL-C lowering is a major impediment to the primary and secondary prevention of ASCVD. In a study of US adults between 2007 and 2010 (8), the most reported reasons for using supplements were to “improve” (45%) or “maintain” (33%) overall health. In only 23% of cases were supplements used based on recommendations of a health care professional (8). In a survey of Black US individuals with hyperlipidemia, 78% believed dietary supplements were equally or more effective than prescription statins, and 58% reported using supplements instead of prescription medications (9). A major contributor to this belief is the marketing patterns of these items, with the United States having the largest supplement market in 2020 at 50.1 billion USD annually and China having the second largest market at 18.3 billion USD (10). Some common supplements which are perceived in the community to lower LDL-C and will therefore be the focus of this review include red yeast rice, omega-3 fatty acids, garlic extract, plant sterols, cinnamon, and turmeric. Despite this belief, data to support the clinically significant LDL-C lowering and anti-inflammatory effects of these supplements are lacking; rather, available data suggest that they are actually ineffective in LDL-C lowering (11) and for ASCVD reduction (12, 13).

The aim of this narrative review is to discuss the historical origins, theoretical mechanisms of action, effects on lipid metabolism and inflammatory markers, and association with ASCVD outcomes of common dietary supplements. The review focuses on supplements evaluated in the Supplements, Placebo or Rosuvastatin (SPORT) trial (11), which is one of few high-quality trials investigating dietary supplement use for “cholesterol health.”

## Historical origin-herbal medicines to dietary supplements

**Fig. 1:**
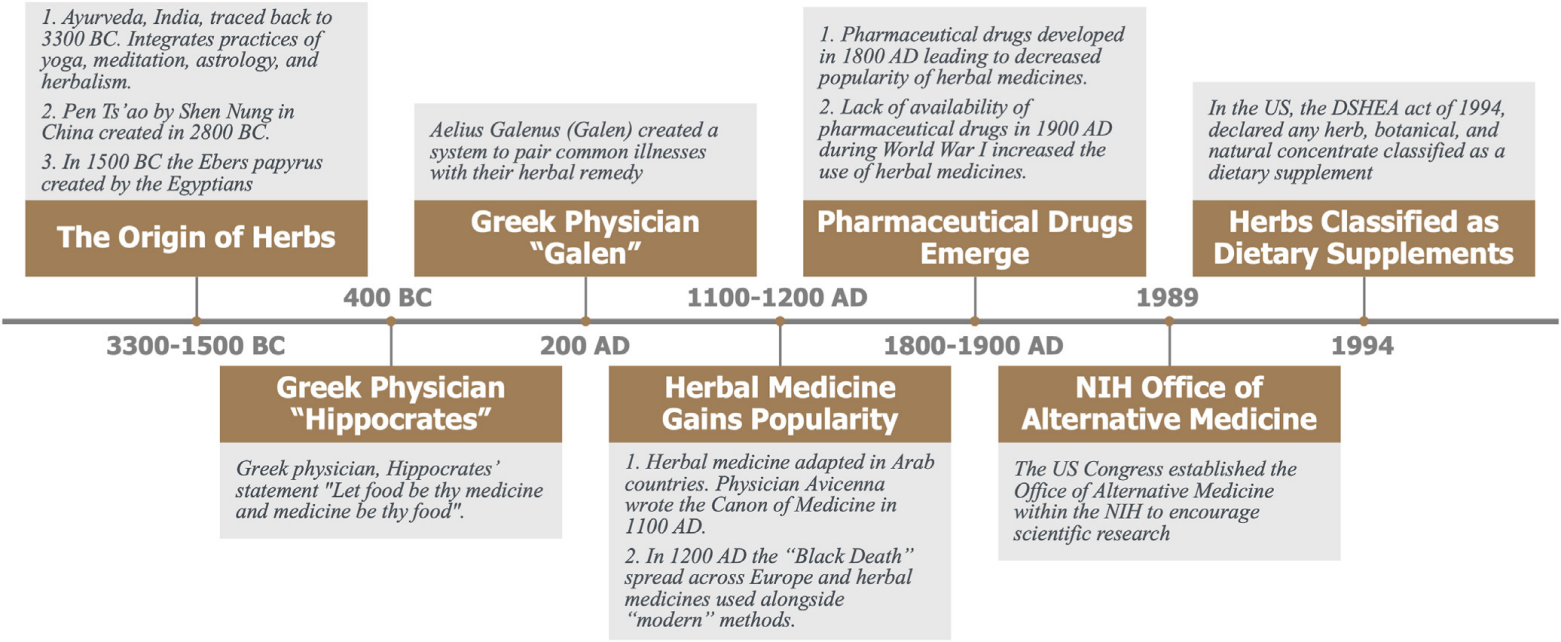
A historical timeline linking dietary supplements to the origin of herbal medicine.

The World Health Organization defines traditional medicine as therapeutic practices that have been in existence for hundreds of years before the development and expansion of modern medicine but are still practiced presently (14). Herbal medicine constitutes only those traditional medicines used for therapeutic reasons that are primarily derived from plant preparations such as leaves, bark, flowers, roots, fruits, and seeds. Modern-day pharmaceutical drugs and dietary supplements are linked to historical and cultural events dating back to 3300 BC (Fig. 1). The first written account of the use of herbal medicine came from China in 2800 BC, where the Pen Ts’ao by Shen Nung compiled traditions of agricultural and medicinal plant treatments (15). Next, around 1500 BC the Egyptians created the Ebers papyrus which is a compilation of medical texts that contains over 700 formulas (16). These papers contained an accurate description of how the human circulatory system works, including the existence of blood vessels and the function of the human heart. During the same time, the Ayurveda in India, which translates to the “Science of Life,” encouraged the concept of integrating yoga, medicine, astrology, and herbal medication/supplement for holistic health and well-being (17). By 400 BC, the Greeks documented further use of herbal remedies with Hippocrates, a Greek physician regarded as the father of Western medicine, emphasizing the role of diet, exercise, and happiness as the cornerstones of health (18). This belief formed the foundation of his infamous statement “let food be thy medicine and medicine be thy food.” One such example of Hippocrates’ philosophy, based on the initial discoveries of ancient Egyptians, was the use of extracts of myrtle and willow leaves to treat inflammatory pain and relieve the pain of childbirth. This discovery was later recognized in modern-day medicine as the origin of aspirin use as an anti-inflammatory.

The Roman empire, which was heavily influenced by Greek medical tradition, spread the use of herbal medicines throughout their empire and developed a culture of cultivating herbal medicines by 50 AD. The first classification system that paired common illnesses with their herbal remedy was then prepared by the Greek herbal practitioner, Aelius Galenus (Galen) (15). Herbal medicine made its way to Arab countries, culminating with the creation of the Canon of Medicine, which gave mention to the use of herbal medicines by Physician Avicenna (19). Additionally, Moses Maimonides was a Jewish philosopher turned physician who translated the writings of Hippocrates, Galen, and Aristotle, eventually writing 10 books on the use of nutraceuticals to treat seizures, asthma, and hemorrhoids. Between 1200 and 1800 AD, the “Black Death” spread across Europe and herbal medicines were used alongside “modern” methods such as bleeding, purging, arsenic, and mercury. Due to a large number of untrained pharmacists giving care that was thought to be inadequate, herbal medicine gained further popularity and was endorsed by leaders within the United Kingdom. King Henry VII and the UK parliament promoted and encouraged the use of herbal medicines and herbalists, while preacher Charles Wesley advocated for balanced dietary habits, good hygiene, and herbal treatments for healthy living. During the 19^th^ century, pharmaceutical drugs were being developed and gained popularity, however a lack of availability during World War I led to another increase in the use of herbal medicines. This postwar period saw an expansion in the international pharmaceutical industry and the discovery of penicillin.

In 1989, the US Congress established the Office of Alternative Medicine within the National Institutes of Health to encourage scientific research in the field of traditional and herbal medicine leading to increased investment (20). During that time a common, internationally recognized, and regulatory framework that governed the use of herbal medicine did not exist, however legislative guidance for the use of herbal medicines in individual countries to regulate clinical indications, efficacy, and safety was in place. At that time, what was referred to as “herbal medicines” were generally sold as supplements to food until the passage of the Dietary Supplement Health and Education (DSHEA) Act (1994) in the US (1). Under the DSHEA Act, the nomenclature of any herb, botanical, and natural concentrate, metabolite, and constituent of an extract is now referred to as a dietary supplement.

## The regulation of dietary supplements

The Food and Drug Administration (FDA) does not have the authority to approve dietary supplements for safety and effectiveness or their labeling before they are sold in stores or online (21). Companies are responsible for ensuring that their products are safe and label claims are truthful and substantiated. As such, dietary supplements are not subjected to the same testing, manufacturing, and labeling standards as pharmaceutical drugs. Instead, they are regulated by the DSHEA act (1) which encourages companies to adhere to the FDA’s good manufacturing practice regulations that outline standards by which manufacturers ensure the quality of dietary supplements created for sale. A product being sold as a dietary supplement in the US can explain on its label or in any of its packaging how the supplement may influence different actions in the body but cannot suggest that it can diagnose, treat, prevent, or cure a specific disease or condition without specific approval from the FDA.

Prior to 2010, there were no regulations in place to standardize and ensure the batch-to-batch consistency of the ingredients in dietary supplements, the implications of which include variability in the composition of differing iterations of the same product (20). Under current regulations, the FDA periodically inspects the facilities of dietary supplement manufacturing companies to ensure compliance with manufacturing and labeling standards (i.e., claims made are truthful and substantiated) and monitors adverse event reports made by industry, consumers, and health professionals (22). Despite oversight of manufacturing and labeling standards, the FDA has no quality controls or regulations on supplement dosing or product purity. For example, omega-3 fatty acids are polyunsaturated fats, and many patients take these for their purported cardiovascular benefits in the form of supplements. However, one study demonstrated that of 32 fish oil supplements available for purchase in New Zealand, only three contained quantities of EPA and DHA that were equal to or higher than the labeled content (23). Additionally, only 8% of fish oil supplements met international recommendations. Another study, including three of the top-selling fish oil dietary supplements in the US, reported that fish oil dietary supplements contained more than 30 different fatty acids (24). These findings were not present when the content of prescription omega-3 fatty acids was analyzed.

Standardizing the composition of dietary supplements remains a challenge. While the FDA’s move to periodically inspect manufacturing facilities is a step in the right direction, variability in the batch-to-batch contents of dietary supplements remains a valid concern.

## Common dietary supplements used for the purpose of lipoprotein and lipid-lowering

Several dietary adjuncts are marketed to promote “cholesterol health” by lowering LDL-C and/or triglycerides (TGs). Below we discuss the historical origin, the proposed mechanisms of action, effects on lipoprotein and inflammatory markers, adverse effects, and cardiovascular outcome trial data for some commonly used dietary supplements. As with the recently published studies (11, 25), we reviewed in detail red yeast rice, omega-3 fatty acids, garlic extract, plant sterols, cinnamon, and turmeric (Table 1).

**Table 1:** Properties of commonly used dietary supplements that are believed to be lipoprotein or lipid-lowering

Supplement	Origin	Proposed Mechanism of Action	Participant Characteristics	Dose/Lipid Effects	Anti-inflammatory Effects	Adverse Effects	Data on ASCVD Outcomes	Expert Opinion/Clinical Recommendation
Red yeast rice	Traditional Chinese medicine	Inhibition of HMG-CoA Reductase	• Hyperlipidemia (26) • statin intolerance (26) • ASCVD (26, 27, 28, 29)	At a dose of 1200–4800 mg per day: 14.7%–48.2% ⇓ LDL-C, 15%–46.3% ⇓ TG, and 0%–17.4% ⇑ HDL-C (26)	50% ⇓ hsCRP with weighted mean difference of −0.14 mg/L (27, 28)	• Myalgias • Hepatotoxicity	China coronary secondary prevention study trial showing ASCVD benefit (29)	• Avoid use in favor of statin therapy when indicated.
Omega-3 fatty acids	Potential cardiovascular benefits reported in observational studies	• Anti-inflammatory • Antioxidant • ⇓ TG • Atherosclerotic plaque stability	• Diabetes mellitus (30, 31, 32, 33, 34, 35, 36) • Hyperlipidemia (30, 31, 32, 33, 34, 35, 36) • Hypertension (30, 31, 32, 33, 34, 35, 36) • ASCVD (30, 31, 32, 33, 34, 35, 36)	1. EPA/DHA dose: 0.045–5.9 g/day 2. Fish diet: 0.9 to 3.8 servings per week 3. Plant-derived omega-3 fatty acids: 0.8 g to about 5 g/day Variable LDL-C effect, 15% ⇓ TG, and 7%–12% ⇑ HDL-C (31)	⇔ effect on CRP (46, 32)	• Mild GI disturbance • Mercury contamination • Atrial fibrillation, particularly with higher doses	• ⇓ ASCVD prior to statins (30, 33) • ⇔ ASCVD benefit with statin therapy (34, 35, 36)	• Marine and plant omega-3 fatty acids can be derived from a balanced diet. • For the purposes of lipid-lowering, avoid over the counter fish oils given the lack of benefit and possible risks of atrial fibrillation and bleeding. • Consider icosapent ethyl as an alternative with clinical benefit seen in the REDUCE-IT trial.
Garlic	Culinary agent used in various cultures	Inhibition of HMG-CoA reductase and other enzymes involved with cholesterol synthesis	• Primarily primary prevention (37)	1. Garlic powder: 600-5,600 mg/day 2. Garlic oil: 9–18 mg/day 3. Aged garlic extract: 1,000–7,200 mg/day 4. Raw garlic: 4–10 g/day • 2%–11% ⇓ LDL-C, 5%–11% ⇓ TC • ⇔ effect on HDL-C or TG (37)	⇓ CRP with mean weighted difference of −0.05 mg/dl (47)	• Mild GI disturbance • Increased bleeding risk	Not available	Avoid concentrated use as a supplement. • May be included for spice/flavor as part of a healthy balanced diet, but not for the explicit purpose of lipid lowering and cardiovascular health benefits. • Preferentially use statin therapy when indicated.
Plant sterols	Structurally similar to cholesterol with potential LDL-C effects first identified in 1950	Inhibits intestinal absorption of cholesterol	• Hyperlipidemia (38, 39) • Metabolic syndrome (38, 39) • Obesity (38, 39) • ASCVD (39)	Plant sterol dose 0.19 to 9.0 g/day • 8.3% ⇓ LDL-C • ⇔ effect on HDL-C or TG (38)	⇔ anti-inflammatory effects (39)	No major reported adverse effects	Not available	• May serve as an adjunct to a heart healthy diet. • However, modest lipid lowering so clinicians should take time to set realistic expectations. • Avoid in patients with suspected beta sitosterolemia. • Preferentially use statin therapy when indicated
Cinnamon	• Southeast Asia Spice	• Antioxidant, • Anti-inflammatory ⇓ glycogen synthesis, glycogenolysis, glucose absorption	• Diabetes mellitus (40, 41) • Hyperlipidemia (40, 41) • Obesity (41)	1. Cinnamon powder: 1–6 g of per day 2. Spray-dried water extract of cinnamon: 500 mg per day 3. Ground cinnamon: 20g per day. • 0%–31% ⇓ TG • ⇔ effects on HDL-C or LDL-C (40)	⇓ CRP with weighted mean difference of −0.22 mg/dl (41)	• Mild GI disturbance • Hepatotoxicity	Not available	• Avoid concentrated use as a supplement. • May be included for spice/flavor as part of a healthy balanced diet, but not for the explicit purpose of lipid lowering and cardiovascular health benefits. • Preferentially use statin therapy when indicated.
Turmeric	• Southeast Asia Spice	• Anti-inflammatory • Antioxidant • ⇑insulin sensitivity	• Hyperlipidemia (42, 43, 44, 45) • Diabetes mellitus (42, 43, 44, 45) • Hypertension (42, 43, 44, 45) • Obesity (42, 43, 44, 45)	Purified curcumin or a curcuminoid mixture, extracts with determined content of curcumin or curcuminoids, or turmeric powder, regardless of dosage and frequency • Inconclusive data (42, 43, 44)	⇔ effect on CRP (45)	• Mild GI disturbance	Not Available	• Avoid concentrated use as a supplement. • May be included for spice/flavor as part of a healthy balanced diet, but not for the explicit purpose of lipid lowering and cardiovascular health benefits. • Preferentially use statin therapy when indicated.

ASCVD, atherosclerotic cardiovascular disease; GI, gastrointestinal; TC, total cholesterol; TG, triglyceride.

### Red yeast rice

Red yeast rice, a product of yeast fermentation of white rice, has been used since 800 AD in traditional Chinese cuisine and medicine (48). The fermentation forms several types of monacolins, and fungal metabolites with a chemical structure similar to statins. One subtype, monacolin K, is structurally similar to lovastatin with LDL-C lowering mediated through the reversible inhibition of 3-hydroxy-3-methyl-glutaryl-CoA reductase. (49) However, the amount of monacolin K across red yeast rice products is tremendously variable ranging from none to large amounts. A 2017 analysis of 28 brands of commercially available red yeast rice in the US found two brands contained no monacolin K, while the other 26 brands ranged in content of monacolin K 60-fold from 0.09 to 5.48 mg among the recommended daily serving of 1,200 mg of read yeast rice (50).

A meta-analysis of 20 randomized controlled trials (RCTs) including primary and secondary prevention patients (e.g., hyperlipidemia, statin intolerance, coronary artery disease, diabetes mellitus), who were taking red yeast rice, 1200 mg–4800 mg per day containing 4.8 mg–24 mg monacolin K, (n = 6,663 participants) demonstrated an approximate 14.7%–48.2% reduction in LDL-C (mean difference 39.4 ± 7.2 mg/dl), 15%–46.3% decrease in TG levels (mean difference 23 ± 8 mg/dl) and a 0%–17.4% increase in HDL-C (mean difference 2.7 ± 1.6 mg/dl) compared to placebo (26). When red yeast rice was compared to low/moderate intensity statins (pravastatin 40 mg, simvastatin 10 mg, and lovastatin 20 mg) there was no significant difference in LDL-C lowering, the mean difference of change score between them both was 1.2 ± 1 mg/dl (26). Additional anti-inflammatory effects have been reported, with a 50% decrease, mean 1.4 mg/L lowering, in high-sensitivity C–reactive protein (hsCRP) levels which show a dose-dependent response (27, 28). In previous RCTs, the rates of kidney injury, liver enzyme elevation, and muscle-related symptoms were reported as similar between the red yeast rice and placebo groups (26, 51). However, there is increased reporting of rhabdomyolysis/myopathy in the FDA adverse event reporting system, odds ratio 8.44 [95% CI 5.44–13.10], and hepatic disorders in the Center for Food Safety and Applied Nutrition Adverse Event Reporting System, odds ratio 13.71 [95% CI 5.44–34.57] (52). A safe monacolin level from red yeast rice which does not increase the concern for musculoskeletal or hepatic injury is yet to be determined (53).

While most trials evaluated the effects of red yeast rice over a relatively short period, the China Coronary Secondary Prevention Study trial in 2008 evaluated Xuezhikang, a purified red yeast rice extract, over 4.5 years. This RCT showed that in 4,870 patients with a prior myocardial infarction (MI), 300 mg of Xuezhikang was associated with a lower risk of fatal, hazard ratio (HR) 0.67 [95% CI 0.52–0.88], and nonfatal MI, HR 0.38 [95% CI 0.27–0.54]), cardiovascular death, HR 0.70 [95% CI 0.54–0.89], and all-cause mortality, HR 0.67 [95% CI 0.52–0.82] (29). Xuezhikang was well tolerated during the trial. The most common adverse effect was minor gastrointestinal effects, and mild increases in transaminases, and creatinine kinase, which was similar to that seen in the placebo group (29). In contemporary practice, most patients (>90%) can tolerate at least a moderate-intensity statin (54), the manufacturing of which is under more stringent FDA regulations than red yeast rice. As such, statins are expected to confer greater ASCVD benefits with a more predictable safety profile.

### Omega-3 fatty acids

Omega-3 fatty acids are polyunsaturated fatty acids found in oily fish (marine-derived) such as salmon, mackerel, and sardines, and some plant foods (plant-derived) like walnuts, chia seeds, and flax seeds. Interest in omega-3 fatty acids initially arose when epidemiologic studies in the 1970s found low rates of cardiac disease in Eskimo populations with diets high in oily fish (55). Subsequent observational studies demonstrated associations between a diet high in “fatty fish” and lower risks of coronary artery disease, and elevated serum omega 3 levels, and decreased odds of sudden death (56, 57, 30). At the time, multiple proposed theories existed for the interplay between omega-3 fatty acids and ASCVD including anti-inflammatory and antioxidant effects, improving endothelial function and improving atherosclerotic plaque stability (58, 59). Marine-derived omega-3 fatty acids (combination of EPA/DHA at a dose of 0.045–5.9 g per day) lower TG levels by approximately 15% (net change of −27 mg/dl, 95% CI −20 to −33 mg/dl) on average have modest but varying effects on LDL-C (+6 mg/dl, 95% CI +3 to +8 mg/dl) and increase HDL-C by 7%–12% (+1.6 mg/dl, 95% CI +0.8 to +2.3 mg/dl) (31). Data evaluating the effects of plant-derived omega-3 fatty acids, namely alpha linolenic acid on lipid biochemistry are sparse. The limited studies that do report changes of TG, total cholesterol (TC), LDL-C, and HDL-C levels with plant-derived omega-3 fatty acids are of poor quality and are limited by small sample sizes or considerable confounding (31, 60). The uncertainty related to alpha linolenic acid may be related to the reported anti-inflammatory effects (61), its role in producing DHA and EPA (62) or confounding by measurement errors (erroneous inclusion of other fatty acids). The limited ability of humans to elongate and desaturate plant-derived omega-3 fatty acids to EPA, even when fed at high levels, is hypothesized as a possible explanation for the lack of convincing data supporting plant-derived omega-3 fatty acids considerably altering TG, TC, LDL-C, and HDL-C levels (31). Dietary supplements, of generally lower doses of omega-3 fatty acids (∼1 g/day), have not been consistently shown to reduce inflammatory markers (31, 63). At high doses of EPA such as those seen in the use of icosapent ethyl (4 g/day), a prescription omega-3 fatty acid, omega-3 supplements are associated with lower hsCRP levels (46, 32). The most common adverse effect observed is mild gastrointestinal disturbances, while the most concerning complication from a clinical perspective is the risk of atrial fibrillation, especially at doses greater than 1 g per day (64, 65).

Trials of omega-3 fatty acid supplements (850–882 mg EPA and DHA in the average ratio of EPA/DHA 1:2) prior to the use of statin therapy suggested some ASCVD benefit, with a HR 0.85 [95% CI 0.74–0.98] for death, nonfatal MI or stroke, in patients with a prior MI (30, 33). However, subsequent trials investigating the use of omega-3 supplements in patients already taking statin therapy demonstrated no ASCVD benefit (34, 35, 36). Similarly, the VITAL trial evaluated the use of marine omega-3 fatty acids (combination DHA 380 mg and EPA 460 mg) in a primary prevention cohort of men ≥50 and women ≥55 years old and showed no difference in the incidence of major cardiovascular events (HR, 0.92; 95% CI, 0.80 to 1.06; *P* = 0.24) (66). A 2019 umbrella review (12) demonstrated that dietary supplementation with omega-3 long-chain polyunsaturated fatty acids was associated with reduced risk for MI (relative risk, 0.92 [CI, 0.85 to 0.99]) and coronary heart disease (relative risk, 0.93 [CI, 0.89 to 0.98]). However, the quality of evidence as assessed by the Grading of Recommendations Assessment, Development, and Evaluation approach was deemed “low-certainty.” Most of these studies evaluating the use of omega-3 fatty acid supplements contained a combination of EHA and DHA in differing proportions. In 2007, the JELIS trial (67) randomized 9,326 patients to 1800 mg of EPA daily [capsules that contained 300 mg of highly purified (>98%) EPA ethyl ester (Mochida Pharmaceuticals, Tokyo, Japan)] with statin therapy (pravastatin 10–20 mg or simvastatin 5–10 mg) and 9319 patients to statin-only therapy (pravastatin 10–20 mg or simvastatin 5–10 mg) with a 5-year follow-up (Table 2). The authors reported a significant reduction in any major coronary event (2.8% in the EPA group vs. 3.5% in the statin-only group, *P* = 0.011) independent of LDL-C lowering. On this basis, the REDUCE-IT trial (68) evaluated prescription-grade EPA (icosapent ethyl) at a dose of 4 g/day compared to a mineral oil placebo and demonstrated ASCVD benefit in patients with established ASCVD or with diabetes and other risk factors, who had been receiving statin therapy and who had a fasting TG level of 135–499 mg/dl (HR, 0.75; 95% CI 0.68 to 0.83; *P* < 0.001). The number needed to treat to avoid one primary end-point event (a composite of cardiovascular death, nonfatal MI, nonfatal stroke, coronary revascularization, or unstable angina) over 4.9 years was 21. However, higher rates of atrial fibrillation (3.1% *vs.* 2.1%, *P* = 0.004) and possibly serious bleeding (2.7% *vs.* 2.1%, *P* = 0.06) were noted. The EVAPORATE trial (n = 80) (69) sought to add important mechanistic data to the beneficial effects of icosapent ethyl compared to a mineral oil placebo by showing significant regression of low-attenuation coronary artery plaque volume on multidetector computed tomography compared with placebo over 18 months. Nevertheless, similar to that seen in prior studies of omega-3 supplements (64, 65), a major adverse effect of icosapent ethyl was the higher risk of atrial fibrillation (5.3% in the icosapent ethyl arm *vs.* 3.9% in the placebo group, *P* = 0.003) (68). On the other hand, the STRENGTH trial (n = 13,078) sought to evaluate the effects of 4 g per day of a carboxylic acid formulation of EPA and DHA (omega-3 CA) on cardiovascular outcomes in patients with atherogenic hyperlipidemia and high cardiovascular risk. When compared to placebo (corn oil), there was no significant difference in the composite of cardiovascular death, nonfatal MI, nonfatal stroke, coronary revascularization, or unstable angina requiring hospitalization (12% with omega-3 CA *vs.* 12.2% with corn oil, *P* = 0.84) (70). This trial reported a higher rate of gastrointestinal disturbances as an adverse effect (24.7% *vs.* 14.7%, *P* was statistically significant). Controversy exists over the conflicting results seen in REDUCE-IT and STRENGTH trials and whether they are related in part to detrimental effects of the mineral oil placebo in REDUCE-IT or different formulations of omega-3 studied. The OMEMI trial compared the effect of 930 mg EPA and 660 mg DHA to placebo (corn oil) in elderly patients with a recent MI (71). There was no difference in the composite cardiovascular outcome of nonfatal MI, revascularization, stroke, all-cause death, and heart failure hospitalization. There was no significant difference in the rate of bleeding between groups while incidence of new onset atrial fibrillation was borderline higher in the omega-3 group (7.2% *vs.* 4.0%, *P* = 0.06).

**Table 2:** Comparison of recent trials investigating the effect of prescription omega-3 fatty acids on cardiovascular outcomes

Study Name	Year	Sample Size	Follow-Up (years)	Population	Intervention	Control	Primary Endpoint	Adverse Effects
JELIS (67)	2007	18,645	4.6	Hyperlipidemia (LDL-C ≥170 mg/dl)	EPA 600 mg TID^a^ + statin^b^ (open label)	Statin^b^	↓ Major coronary events (2.8% *vs.* 3.5%, *P* = 0.011)	↑ Gastrointestinal disturbance (3.8% *vs.* 1.7%, *P* < 0.0001) ↑ Skin rash (1.7% *vs.* 0.7%, *P* <0.0001) ↑ Bleeding (1.1% *vs.* 0.6%, *P* <0.0006)
REDUCE-IT (68)	2019	8,179	4.9	ASCVD or diabetes and other risk factors, on statin therapy + TG level 135–499 mg/dL	Icosapent ethyl 2G BID (Highly purified EPA)	Mineral oil	↓ Composite primary cardiovascular outcome HR, 0.75; 95% CI 0.68 to 0.83; *P* < 0.001	↑ Atrial fibrillation or flutter (3.1% *vs.* 2.1%, *P* = 0.004) ↑ Serious bleeding (2.7% *vs.* 2.1%, *P* = 0.06)
VITAL (66)	2019	25,871	5.3	Primary prevention cohort of men ≥50 years of age and women ≥55 years of age	Marine omega-3 fatty acid 1G per day (combination EPA/DHA)Ψ	Olive oil	⇔ In major adverse cardiac events (a composite of myocardial infarction, stroke, or death from cardiovascular causes). Hazard ratio, 0.92; 95% CI, 0.80 to 1.06; *P* = 0.24.	⇔ in gastrointestinal symptoms, major bleeding episodes, or other serious adverse events
EVAPORATE (69)	2020	80	1.5	Coronary atherosclerosis by MDCT, on statin therapy and TG level 135–499 mg/Dl	Icosapent ethyl 2G BID (Highly purified EPA)	Mineral oil	↓ In coronary low attenuation plaque volume (−17% *vs.* +109%, *P* = 0.0061)	↑ Atrial fibrillation (5.3% *vs.* 3.9%, *P* = 0.003)
STRENGTH (70)	2020	13,078	3.4	High cardiovascular risk, hypertriglyceridemia, and low levels of HDL-C (LDL-C ≤100 mg/dl or TG 180–500 mg/dl and HDL-C < 42 mg/dl for men and < 47 mg/dl for women)	Omega-3 carboxylic acid 4G Daily. (combination EPA/DHA)	Corn oil	⇔ Difference in the composite primary cardiovascular outcome (12.0% *vs.* 12.2%, *P* = 0.84)	↑ Gastrointestinal disturbance (24.7% *vs.* 14.7%, *P* < 0.05) ↑ Atrial fibrillation (HR 1.69, 95% CI 1.29–2.21, *P* < 0.001)
OMEMI (71)	2020	1,027	2.0	Patients aged 70–82 years with recent (2–8 weeks) acute myocardial infarction	Combination EPA 930 mg, DHA 660 mg 1800 mg Daily	Corn oil	⇔ Composite of nonfatal AMI, unscheduled revascularization, stroke, all-cause death, heart failure hospitalization (21.4% *vs.* 20.0%, *P* = 0.60)	Borderline ↑ in atrial fibrillation (7.2% *vs.* 4.0%, *P* = 0.06) ⇔ major bleeding (10.7% *vs.* 11.0%, *P* = 0.87)

Ψ 1 g per day as a fish-oil capsule containing 840 mg of omega-3 fatty acids, including 460 mg of EPA and 380 mg DHA.

a Capsules that contained 300 mg of highly purified (>98%) EPA ethyl ester (Mochida Pharmaceuticals, Tokyo, Japan).

b Pravastatin 10–20 mg or simvastatin 5–10 mg.

Based on the data available, in general omega-3 fatty acid supplements modestly lower or in some cases increase LDL-C with no proven ASCVD benefit over statin therapy. However, the prescription-grade pure EPA formula (icosapent ethyl) has been shown to lower residual ASCVD risk in patients already taking maximal statin therapy. Unlike dietary supplements, this form of omega-3 fatty acid requires a prescription and is subject to regulatory oversight (67, 68, 70).

### Garlic

Historically in various cultures, garlic has been used for culinary and medicinal purposes, ranging from improving circulation to aiding digestion (72). Garlic is purported to exert its LDL-C and TG-lowering effects by inhibiting 3-hydroxy-3-methyl-glutaryl-CoA reductase and other enzymes involved in cholesterol synthesis (73, 74). RCTs of garlic supplementation on LDL-C, HDL-C, and TGs have produced unconvincing results. One meta-analysis with a baseline TC of 200 mg/dl and LDL-C of 130 mg/dl showed that garlic supplementation had a modest lowering of LDL-C, mean reduction of −6.4 mg/dl, 95% CI −1.1 to −11.8 mg/dl (2%–11%), and TC, mean reduction of −15.3 mg/dl, 95% CI −9.8 to −20.8 mg/dl (5%–11%), without a significant effect on HDL-C or TGs (37). Pertaining to anti-inflammatory effects, garlic is associated with lower CRP levels with one meta-analysis showing a weighted mean difference of −0.053 mg/dl compared to placebo (47). Generally, garlic is well tolerated with the most common side effects being “garlic odor” and gastrointestinal symptoms including nausea, heartburn, bloating, abdominal pain, and diarrhea (75). Additionally, prolonged clotting time and surgical bleeding complications have been reported in case reports of patients taking high doses of garlic supplements (75).

Currently, there are no trials evaluating the effect of garlic supplements on ASCVD outcomes.

### Plant sterols

Sterols are critical components of the cell membrane that regulate membrane fluidity, permeability, and function. The primary sterol in humans is cholesterol, whereas there are a variety of different plant sterols that are structurally similar but distinct. The first study to evaluate the LDL-C lowering effects of plant sterols was published in the 1950s (76). Plant sterols are effective by inhibiting intestinal cholesterol absorption (77). A meta-analysis of 114 RCTs compared the dose-response relationship of plant sterols with placebo in patients with a mean baseline LDL-C of 147 ± 23.2 mg/dl. LDL-C was lowered by approximately 8.3%, with a mean reduction of 15.9 mg/dl (95% CI 13.5–18.6 mg/dl), with no significant effects on HDL-C or TGs (38). Of note, in this meta-analysis, if the active treatment arm received plant sterols plus a statin, the placebo group had to have received the statin to permit isolation of the independent effects of the plant sterols on LDL-C. However, plant sterols have not been consistently demonstrated to reduce inflammatory markers (39). Plant sterols are well tolerated and significant adverse effects have not been reported (78).

Currently there are no RCTs evaluating the effects of plant sterols on ASCVD outcomes. Plant sterols may serve as an adjunct to heart-healthy dietary habits; however, clinicians should ensure that patient expectations regarding the extent of LDL-C lowering are realistic. Additionally, plant sterols should be avoided in patients with beta sitosterolemia (79), a rare autosomal recessive disorder leading to high concentrations of plant sterols in blood. In these patients, elevated plant sterols have been associated with increased atherosclerosis risk.

### Cinnamon

Cinnamon is a spice made from the inner bark of trees scientifically known as Cinnamomum. The spice originates from Southeast Asia and has been used in South Asian cuisines and as part of traditional medical practices. Cinnamon is advertised as an antioxidant with anti-inflammatory properties and as having cardiovascular and glycemic benefits. Phenolic compounds, such as those found in cinnamon, are thought to reduce oxidative stress and inflammation with additional proposed mechanisms of action, including decreased glycogen synthesis, glycogenolysis, and glucose absorption (40). One meta-analysis of cinnamon supplements in patients with type 2 diabetes mellitus (13 RCTs, 750 participants) showed that cinnamon had no significant effect on LDL-C and HDL-C but modestly lowered TG levels (14.1%) from a baseline of 169.9 mg/dl with a mean weighted difference of −23.91 mg/dl (95% CI −12.4 to −34.5 mg/dl) (40). Cinnamon is also reported to lower CRP levels mean weighted difference of 0.22 mg/dl than placebo (41). The spice is generally well tolerated with the most common side effect being mild gastrointestinal symptoms (80). At high doses, cinnamon is linked with hepatic injury (80). This appears to be driven by the presence of coumarin, a compound found in cinnamon. The tolerable daily intake of coumarin is reported to be 0.1 mg/kg (81), however levels of coumarin in cinnamon have been found to range from undetectable to as high as 8.79 mg/g, with an average of 2.41 mg/g (82). Assuming an average coumarin content of 2.4 mg/g, a 60 kg adult would likely exceed the tolerable daily intake of coumarin by consuming 248 mg of cinnamon. For context, supplements usually contain hundreds or thousands of milligrams of cinnamon.

There are no RCTs on the effect of cinnamon supplements on ASCVD outcomes.

### Turmeric

Turmeric is a yellow-colored spice originating from Southeast Asia that has been used for more than 4,000 years for culinary purposes and as a part of traditional Chinese, Ayurvedic, and Iranian medicine (83). Turmeric’s proposed mechanism of action includes reducing inflammation, acting as an antioxidant, improving insulin sensitivity, and reducing plasma-free fatty acids (84). Trials evaluating the effect of turmeric on lipid parameters show significant heterogeneity in baseline characteristics, the dose of turmeric used, comparison groups, and methods of statistical analysis (85). As such, there is discordant reporting of the effects of turmeric on various lipid parameters (42, 43, 44). For example, one meta-analysis of seven RCTs (n = 649) reported lowering of LDL-C (standardized mean difference = −0.34, 95% CI: −0.53 to −0.15, *P* < 0.0001) and TG (standardized mean difference = −0.21, 95% CI: −0.37 to −0.06, *P* = 0.007) with no effect on HDL-C (42). This study was limited by significant heterogeneity (I^2^ = 73.8%) amongst the included studies and as such the authors applied a random effects model to compensate after which the lowering of TC was no longer significant (*P* = 0.054) (42). Another meta-analysis of findings (10 studies cumulatively including 133 patients taking turmeric and 90 taking placebo) did not indicate a significant effect of turmeric on any lipid parameters [TC 9 (95% CI: −4.6 to +22.5 mg/dl), LDL-C 16.1 (−4.4 to +36.7 mg/dl), TG −1.3 (−9.1 to 6.5 mg/dl) and HDL-C −0.6 (−1.7 to 0.5 mg/dl)] (43). Turmeric is not associated with significant reductions in serum inflammatory markers (45). The most common adverse effects reported with turmeric supplementation are mild gastrointestinal symptoms such as abdominal pain and nausea (42).

There are no RCTs on the effect of turmeric supplements on ASCVD outcomes.

## The SPORT trial

**Fig. 2:**
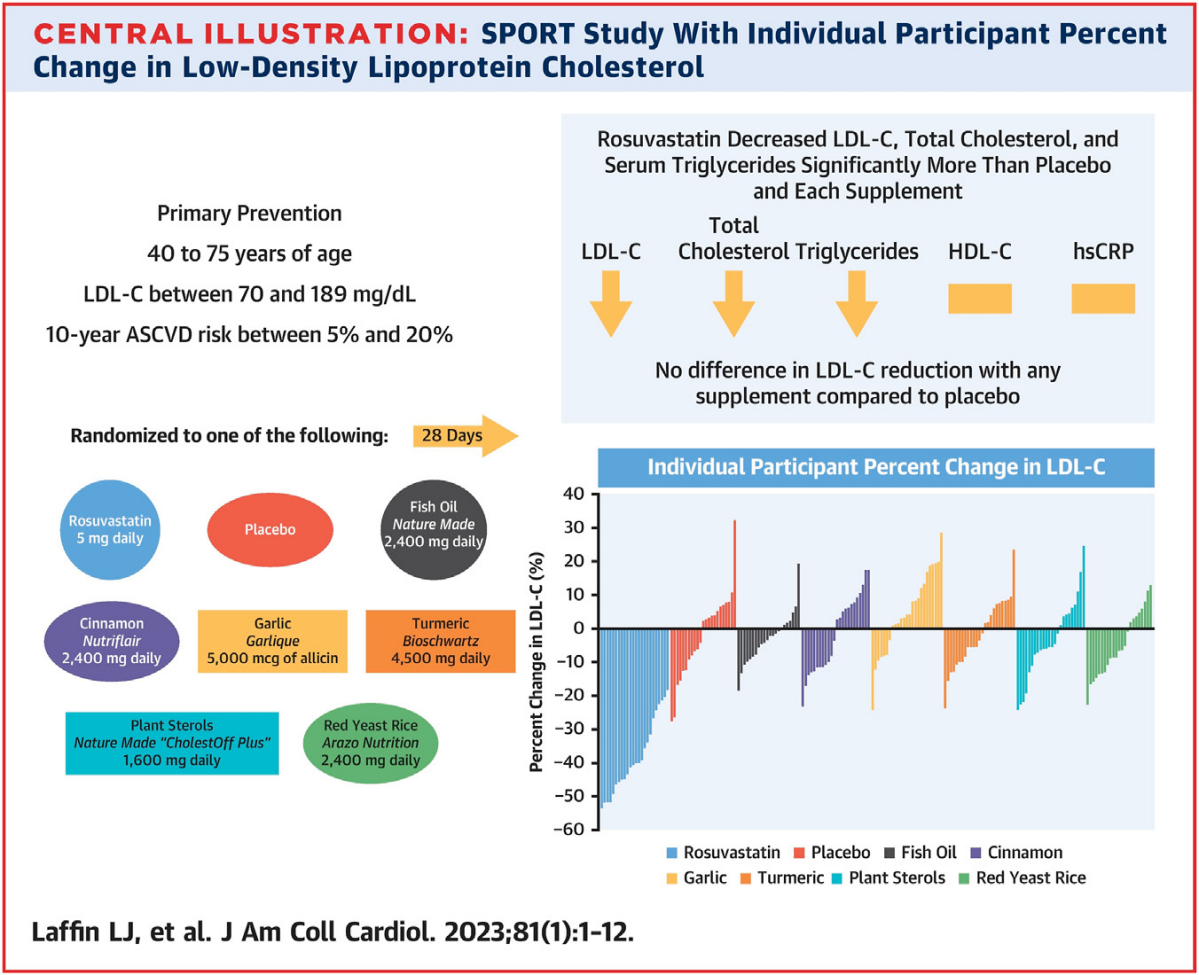
Summary and Central Illustration of the SPORT Trial with individual participant percent change in LDL-C. ∗Adapted from Laffin LJ, Bruemmer D, Garcia M, *et al.* comparative effects of low-dose rosuvastatin, placebo, and dietary supplements on lipids and inflammatory biomarkers. Journal of the American College of Cardiology. 2023;81(1):1–12. DOI: https://doi.org/10.1016/j.jacc.2022.10.013.

The SPORT trial (11) was a single-center, prospective, randomized trial of 190 participants at the Cleveland Clinic Foundation that compared the effects of rosuvastatin 5 mg daily, with placebo and six dietary supplements (red yeast rice, fish oil, garlic, plant sterols, cinnamon, turmeric) on lipid and inflammatory markers (hsCRP) after 28 days of follow-up. Patients enrolled included individuals with no history of ASCVD, baseline LDL-C levels of 70–189 mg/dl, and at borderline to intermediate 10-year risk of ASCVD (5%–20%), and who were not taking statins or other LDL-C lowering therapies. SPORT showed (Fig. 2) that the percent LDL-C reduction with rosuvastatin 5 mg/day was greater than all supplements and placebo (*P* < 0.001), with the difference in LDL-C reduction with rosuvastatin compared with placebo being −35.2% (95% CI: −41.3% to −29.1%; *P* < 0.001). None of the six dietary supplements demonstrated a significant decrease in LDL-C compared with placebo, and the garlic supplement increased LDL-C compared to placebo. Adverse event rates were similar across all study groups.

The SPORT trial included commonly available supplements purchased by consumers and likely reflected exposure to popular supplements that are marketed to patients in everyday life. In contrast to some of the studies discussed above, the SPORT trial did not demonstrate statistically significant reductions in LDL-C with the use of red yeast rice, fish oils, and plant sterols. Furthermore, contrary to previous studies, the SPORT trial showed a small absolute increase in LDL-C with garlic supplement use. The changes in LDL-C with supplement use were modest and not significantly different when compared with placebo use, in stark contrast to the potent LDL-C reduction achieved with a low dose of a high-intensity statin (rosuvastatin 5 mg/dl). The findings of the SPORT trial when compared to prior studies may be due to a difference in study design and quality but may also reflect heterogeneity in the content of supplements. As discussed above, exact contents of dietary supplements may differ from what is claimed on the label.

## Clinician’s perspective

**Fig. 3:**
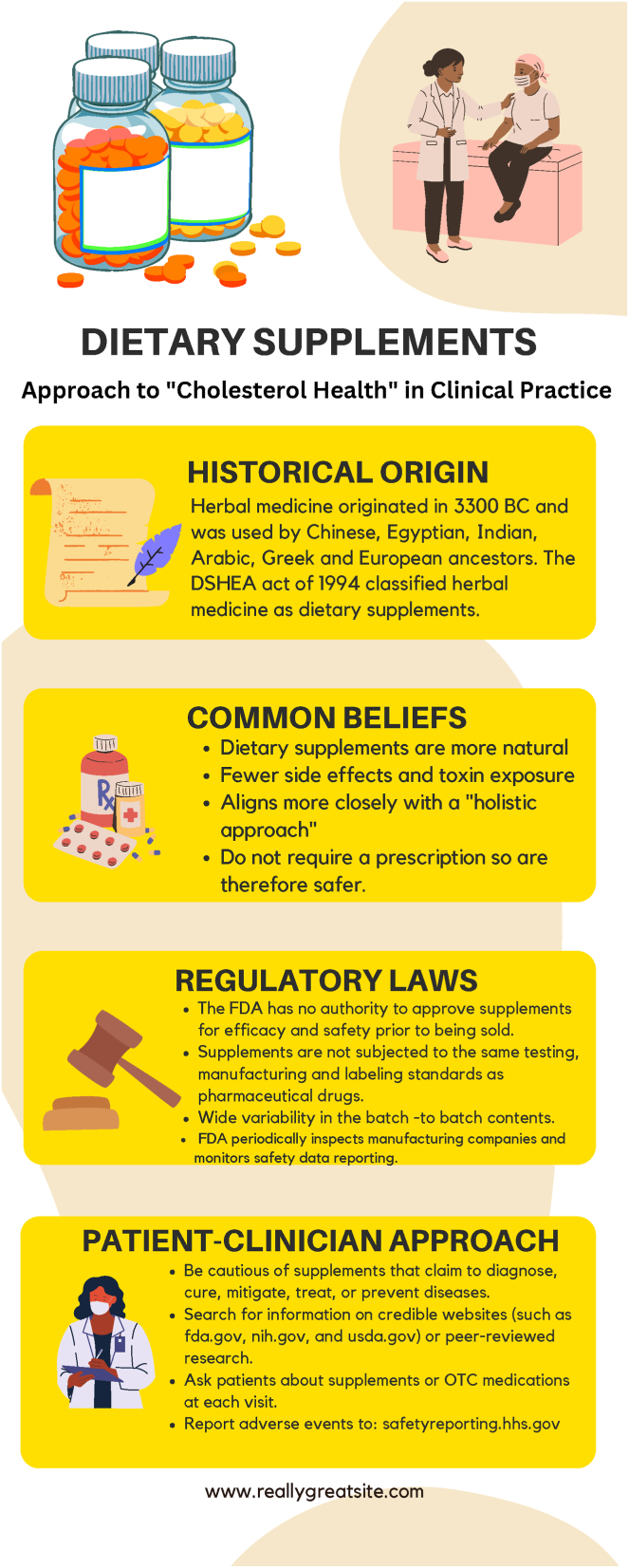
A summary of the historical origin, common beliefs, regulatory laws, and potential approach to the use of dietary supplements for cholesterol health.

Clinicians should consider cultural practices, individual health beliefs, available evidence, and potential adverse effects when evaluating dietary supplement use in patients who are eligible for or are taking evidence-based lipoprotein or lipid-lowering medications (Fig. 3). The holistic approach to lowering ASCVD risk makes dietary supplements an attractive option to many patients, however, their use should not come at the expense of established therapies. Although there are some supportive studies, the evidence is mixed and the highest quality contemporary literature (e.g., SPORT trial) does not show the benefits of commonly used dietary supplements. These supplements are not subjected to the same manufacturing scrutiny as pharmaceutical drugs and as such, the exact contents and level of ingredients in each of these supplements may vary. While most supplements appear to be well tolerated with mild gastrointestinal side effects being most common, supplements such as omega-3 fatty acids increase the risk of atrial fibrillation, garlic is associated with higher bleeding risks, and red yeast rice, given its similarity to statins, is associated with muscle symptoms and hepatic injury.

Lifestyle modifications, including diet and exercise, are the foundation of prevention. When patients express interest in dietary supplements, it is an opportunity to capitalize on patients’ enthusiasm and motivation for doing something “natural” and reinforce the natural benefits of a heart-healthy lifestyle. Furthermore, the use of the pooled cohort equations with a clinician-patient discussion on statin initiation is an evidence-based approach to cholesterol-lowering for ASCVD prevention. Given the strong data to support the use of statins and other proven therapies for dyslipidemia (ezetimibe, PCSK9 inhibition, bempedoic acid and icosapent ethyl) for ASCVD risk reduction, a clinician-patient discussion should always evaluate for the use of dietary supplements and help guide our patients to what we know works.

## Conflict of interest

The authors declare that they have no conflicts with manufacturers of supplements. E. D. M. and S. S. M. declare consulting/advisory board roles with pharmaceutical companies in the lipid field as follows. E. D. M. reports consulting/advisory board roles with Amgen, AstraZeneca, Boehringer Ingelheim, Edwards Lifescience, Esperion, Medtronic, Merck, New Amsterdam, Novartis, Novo Nordisk, and Pfizer. S. S. M. reports consulting/advisory board roles with Amgen, AstraZeneca, BMS, Kaneka, New Amsterdam, Novartis, Novo Nordisk, Sanofi, and 89bio.
